# Evaluation of anthropometric indices and their relationship with maternal nutritional literacy and selected socio-economic and demographic variables among children under 5 years old

**DOI:** 10.1186/s13052-022-01327-1

**Published:** 2022-07-30

**Authors:** Mina Maheri, Maryam Bidar, Hamidreza Farrokh-Eslamlou, Ali Sadaghianifar

**Affiliations:** 1 Reproductive Health Research Center, Clinical Research Institute, Urmia University of Medical Sciences, Urmia, Iran; 2 Department of Public Health, School of Public Health, Urmia University of Medical Sciences, Urmia, Iran; 3 Health System Research Unit, Health Center of Urmia, Urmia University of Medical Sciences, Urmia, 5756115198 Iran

**Keywords:** Anthropometric indices, Maternal nutritional literacy, Children under 5 years old

## Abstract

**Background:**

Considering the destructive effects of malnutrition on the growth, development, and health of children and the importance of identifying the factors affecting it, the present study aimed to investigate the status of anthropometric indices and their relationship with maternal nutritional literacy and selected socio-economic and demographic variables among children under 5 years old.

**Methods:**

This cross-sectional study was conducted on 405 mothers with children under 5 years old in Urmia, Iran. The data collection tool consisted of two parts. The first part was the demographic and socio-economic information of mother and child and the second part was the Evaluation Instrument of Nutrition Literacy on Adults; EINLA.

**Results:**

There was statistically significant relationship between maternal nutritional literacy with weight-for-age, height-for-age, and weight-for-height indices; between weight-for-age index with maternal education, gestational weight gain, and mean weight, as well as mean height of the mother; between weight-for-age and weight-for-height indices with child gender, type of milk consumed, status of starting complementary foods, and history of acute respiratory infection, as well as diarrhea; and finally between height-for-age with family income status.

**Conclusions:**

It is suggested that mothers with low nutritional literacy, mothers whose gestational weight gain was not normal, children whose family income is low, boy child, children with a history of disease, children who consume powdered milk and children who have not started complementary foods at the right time be given priority when designing and implementing educational interventions to enhance nutritional status and anthropometric indices of children.

## Background

Malnutrition of the under-nutrition type is one of the most important health problems, especially in developing countries, whose consequences can be observed in all stages of life from embryonic - to - adult [1]. All ages are at risk of malnutrition and complications arising from it, but the risk among under 5 years old children is most important [1].

Nutrition shortages in children are associated with vast range of complications, including physical and mental retardation, reduction of learning and teachable power, loss of ability, and inability to acquire skills, deduction in resistance to diseases, and even mortality. These conditions will not be reversible in the following years, even with adequate nutrition or more care, improving living conditions and education and it can irrecoverably endanger future generations, as well as national, social, economic and cultural development of a country [1–3].

In developing countries such as Iran, malnutrition due to excessive food intake (overnutrition), along with undernutrition, has put different sectors of society at risk, so that these countries encounter a nutritional transition. The prevalence of overweight and obese also has a range of physical, psychological and social complications for children [4].

Traditionally, the state of child malnutrition in a region is determined by measuring weight, height, and age. The most important indices that come from these sizes are underweight (low weight-for-age), wasting (low weight-for-height), stunting (low height-for-age), and overweight (more weight-for-height). The periodic comparison of anthropometric indices is a good information source as a reference to policy makers and executive directors of the country and the provinces in the field of health [5, 6].

According to the mentioned cases, it can be concluded that one of the important goals of care among children is to improve the anthropometric indices and eliminate malnutrition among them. Achieving this goal requires the design and implementation of a variety of interventions, especially nutrition training. To increase the effectiveness of these interventions, we must first examine and evaluate the malnutrition status and anthropometric indices of children, so that we can focus our interventions more on the dimensions that are problematic.

Another point that should be considered when designing and implementing interventions related to improving anthropometric indices and eliminating child malnutrition is to identify the factors that affect these indices. By identifying and integrating these influential factors in interventions designed and implemented to improve anthropometric indices and eliminate child malnutrition, we can help increase the effectiveness of such interventions.

Among the factors that affect children anthropometric indices is the level of maternal nutritional literacy (MNL) [7–9]. Nutrition literacy is defined as, “the degree to which individuals have the capacity to obtain, process, and understand nutrition information and skills needed in order to make appropriate nutrition decisions” [10]. The findings of the studies show that maternal nutritional literacy is an important factor affecting the nutritional status of children and it has to be considered in all efforts aimed at improving the anthropometric indices and eradicating malnutrition among the children [7–9].

According to the destructive effects of malnutrition on the growth, development and health of children and the lack of studies on the relationship between maternal nutritional literacy with the anthropometric indices of less than 5 years old children in Iran, the present study aims to investigate the relationship between the maternal nutritional literacy with anthropometric indices in less than 5 years old children of Uremia. The findings of this study by determining the nutritional status and anthropometric indices of children and weaknesses in this area, as well as to identify some of the factors affecting these indices, will provide the opportunity and ability to policy makers and health administrations to design and implement different interventions, including nutrition education according to the needs of these children.

## Methods

The present study is a descriptive - analytical cross - sectional study aimed at determining the relationship between the maternal nutritional literacy with anthropometric indices of children less than 5 years old in 2020. The study population was all mothers with less than 5 years old children who referred to health care centers of Urmia. The inclusion criteria for the study include: The mother who has less than 5 years old child, the mother who is the main caregiver of her/his child, the mother’s age range is between 18 and 50 years old, the mother’s education is at least Middle school level, and the absence of congenital malformations and specific disease in the children of under study. The exclusion criteria was the incomplete completion of the questionnaire.

According to the previous similar studies and the prevalence of 50% for Underweight/stunting [8, 11], 95% confidence interval, margin of error 0.05, and using the Sample size formula for estimating a population proportion, the minimum sample size required was determined 385 people. Finally, the sample size was considered 405 people, with probability of loss of 5% of samples and increasing the study power.

$$n=\frac{{z^2}_{\left(1-\frac{\alpha }{2}\right)}P\left(1-P\right)\kern0.5em }{d^2}$$  $$=\frac{(1.96)^2\begin{array}{cc}0.5\left(1-0.5\right)& \end{array}}{0.05^2}=385$$

For sampling, multi-stage cluster sampling method was used. First 10 centers were selected randomly from the comprehensive health services centers of Urmia city, so that there was at least one center from different regions in terms of socio-economic status (high, middle, and low). Then, from each selected center, about 40 mothers with children under 5 years old, among the mothers who met the inclusion criteria and were willing to participate in the study, were entered into the study using convenience sampling method.

The questionnaire used in the present study consisted of two parts. The first part was related to demographic and socio-economic characteristics of mother and child. The second part was the Persian version of Evaluation Instrument of Nutrition Literacy on Adults; EINLA [10], that it was translated by Hemati et al., in Iran and its validity and reliability were measured [12].

The questionnaire contained 35 items by 5 dimensions General nutritional information (10 items), for example, which of the following is essential for dental health?; Reading comprehension and interpretation (6 items), in this dimension, first a short text is presented and the person answers the questions related to that text; Food groups (10 items), in this dimension, ten pictures related to food are presented and the person places them in the relevant food group. Serving sizes determination (3 items), for example, how many servings of milk and dairy products should be consumed daily?; and Read food labels and ability to do simple calculations (6 items), in this dimension, the ability to calculate and evaluate body mass index (BMI), as well as the ability to calculate the amount of calories and other items on a food label are examined. In the study of Hemati et al., the qualitative content validity (expert’s panel) was used to determine the validity of the questionnaire, and the method of calculating Cronbach’s alpha coefficient to determine the reliability. Cronbach’s alpha coefficient was obtained 0.73 for the total questionnaire, which showed the appropriate reliability of the tool [12]. Each question has only one correct answer. While each correct answer was given one point, unanswered or incorrectly answered items were given 0 points. The highest and lowest scores that people achieve from this questionnaire are between 0 and 35. When nutrition literacy level was graded, a total score between 0 and 11 was considered as insufficient, between 12 and 23 as borderline and between 24 and 35 as sufficient [10, 12]. This questionnaire was completed by mothers as a self - report and with the guidance of the researcher.

The weight of children less than 2 years old was measured with the baby scale and the weight of children over 2 years old was measured standing up using other types of medical scales available in the health centers (for example portable scales), with minimal clothing, without shoes and according to the same and pre-compiled instructions. The length of children less than 2 years old was measured lying down using an infantometer and height of children over 2 years old standing up using a stadiometer available in the health centers, without shoes and according to the same and pre-compiled instructions.

Then, Z-Score of weight-for-age (WAZ), height-for-age (HAZ) and weight-for-height (WHZ) indices were calculated using the Anthro software version 3.2.2 (Department of nutrition, WHO, Geneva, Switzerland) [4, 13]. Anthro software has the ability to calculate the WAZ, HAZ, and WHZ of each child by comparing the age, weight and height of children with the World Health Organization standard.

According to the standard of the World Health Organization, the prevalence of underweight was calculated according to the percentage of children under five years of age whose WAZ is below − 2 standard deviations of the WHO Child Growth Standards median. Prevalence of Stunting was calculated according to the percentage of children under five years of age whose HAZ is below − 2 standard deviations of the WHO Child Growth Standards median.

Prevalence of Wasting was calculated according to the percentage of children under five years of age whose WHZ is below − 2 standard deviations of the WHO Child Growth Standards median. Prevalence of Overweight was calculated according to the percentage of children under five years of age whose WHZ is above + 2 standard deviations of the WHO Child Growth Standards median [4–6].

The required data in related to other demographic and socio-economic characteristics of the mother and child were obtained through information recorded in their health record or through interviews with the mothers by the researcher.

This study received ethical permission from the Research Ethics Committee of Urmia University of Medical Sciences (IR.UMSU.REC.1398.187). To maintain ethical considerations, sufficient explanations were first given to the participants about the purpose of the study. They were assured that participation in the study is quite voluntary and if reluctant, they can withdraw from the study, as well as their information would be kept confidential by the researcher and the results would only be reported in general. The questionnaire lacked the name and surname, and finally they received informed consent of the questionnaire.

Finally, data obtained in SPSS version 16 and using descriptive statistics (mean, standard deviation, percentage and frequency) and analysis statistics include the Kolmogorov - smirnov test (to check the normality of the data),independent T-test,one-way ANOVA, chi-square, fisher’s exact test, pearson correlation coefficient were analyzed. The results were significant at the statistical level (*p* < 0.05).

## Results

In the present study, 405 questionnaires were completed, 37 questionnaires were removed due to incomplete information, and finally the data obtained from 368 mothers with children less than 5 years old were analyzed (90.86% response rate). The mean age of under study mothers was 31.46 ± 6.05 years and all married. The majority of mothers were with bachelor’s degree (33.7%), housewife (76.9%), Tork (76.6%), with family income sufficient for living (80.7%), with gestational weight gain between 7 to 12 kg (56.2%), and with gestational age of 37 weeks and more (92.4%). The majority of mothers had reported that they had one child (50.8%). (Table 1).

**Table 1:** Frequency distribution of characteristics of mothers under study

Variables	Categories	n(%)	Variables	Categories	n(%) or
Marital status	Married	368(100)	Family income sufficiency	*Yes*	297(80.7)
Education level	Middle school	77(20.9)		*No*	71(19.3)
	High school	105(28.5)	Gestational weight gain (kg)*	< 7	58(15.8)
	Associate’s degree	32(8.7)		7–12	207(56.2)
				13–16	60(16.3)
	Bachelor’s degree	124(33.7)		> 16	43(11.7)
			Gestational age (week)*	37>	28(7.6)
	Master’s degree	28(7.6)		≥ 37	340(92.4)
	Doctoral degree	2(0.6)	Breastfeeding status*	She is breastfeeding now	153(41.5)
Employment status	Housewife	283(76.9)			
	University/ School student	1(0.3)		She was breastfeeding, but now has stopped breastfeeding	168(45.7)
	government employee	72(19.6)			
				She did not breastfeed at all	47(12.8)
	Self-employed	12(3.2)			
Ethnicity	Tork	282(76.6)	Number of children	1	187(50.8)
	Kord	77(20.9)		2	137(37.2)
	Fars	9(2.5)		3	40(10.9)
**Variables**	**Mean ± SD**			4	4(1.1)
Age (yr)	31.46 ± 6.05				
Height (cm)	162.06 ± 5.31				
Weight (kg)	70.00 ± 11.31				

*n* number, *SD* standard deviation, *yr* year, *kg* kilogram, *cm* centimeter; *,in relation to the child under study

The mean age of under study children was 22.58 ± 16.40 months. The majority of them were boys (52.7%), first child (52.2%), in terms of interval from previous birth 36 months and more (33.7%), and in terms of type of milk consumed in the first 12 months of birth, Breast milk (61.7%). Other information is reported in Table 2.

**Table 2:** Frequency distribution of characteristics of children under study

Variables	Categories	n(%)	Variables	Categories	n(%) or
Age range	0–12	130(35.3)	Exclusive breastfeeding in the first 6-months of life	6 months are not over	48(13.0)
	13–24	112(30.4)			
	25–36	53(14.4)		*Yes*	203(55.2)
	> 36	73(19.8)		*No*	117(31.8)
Gender	Male	194(52.7)	Start receiving complementary foods (month)	Not started	55(14.9)
	Female	174(47.3)		< 4	17(4.6)
				4–6	93(25.3)
Birth order	First	192(52.2)		6	203(55.2)
	Second	140(38.0)	History of acute respiratory infection in the last two weeks	*Yes*	15(4.1)
	Third	32(8.7)		*No*	353(95.9)
	Fourth	4(1.1)			
Interval from previous birth (month)	First birth	192(52.2)	History of diarrhea in the last two weeks	*Yes*	27(7.3)
	< 24	19(5.2)		*No*	341(92.7)
	24–35	33(9.0)	History of intestinal worms in the last two weeks	*Yes*	0(0)
	≥ 36	124(33.7)		*No*	368(100)
Type of milk consumed in the first 12 months of birth	Breast milk	227(61.7)	Hospitalization history in the last 1 month	*Yes*	3(0.8)
				*No*	365(99.2)
	Powdered milk	70(19.0)	Hospitalization history in the last 3 month	*Yes*	7(1.9)
				*No*	361(98.1)
	Combination	71(19.3)	**Variables**	**Mean ± SD**	
Currently consumes breast milk	*Yes*	157(42.7)	Age (month)	22.58 ± 16.40	
	*No*	211(57.3)			
			Weight (kg)	11.44 ± 4.19	
Currently consumes powdered milk	*Yes*	75(20.4)	Height (cm)	82.69 ± 15.34	
	*No*	293(79.6)	birth (gr) Weight at	3178.85 ± 443.68	
			Height at birth (cm)	49.78 ± 1.93	
Currently consumes cow milk	*Yes*	160(43.5)	Head circumference at birth (cm)	34.65 ± 1.31	
	*No*	208(56.5)			

*n* number, *SD* standard deviation, *yr* year, *kg* kilogram, *gr* gram, *cm* centimeter

Based on the findings of the present study, among studied children less than 5 years old, the prevalence of wasting (6%), overweight (9.2%), underweight (4.3%), and stunting (7.9%), were obtained (Table 3).

**Table 3:** Frequency distribution of children under study according to anthropometric indices

anthropometric indices	Subcategories		Z-score	n (%)
weight-for-height (WHZ)	Wasting		≤ −2	22(6.0)
	Normal		> − 2 to + 2 <	312(84.8)
	Overweight		≥ + 2	34(9.2)
weight-for-age (WAZ)	Underweight	Yes	≤ −2	16(4.3)
		No	> −2	352(95.7)
height t-for-age (HAZ)	Stunting	Yes	≤ −2	29(7.9)
		No	> −2	339(92.1)

*n* number

According to the study findings, there was statistically significant relationship between WAZ with mother’s educational level and gestational weight gain, so that the prevalence of underweight in among children whose mothers had a college education compared to children whose mothers had no college education and among the children whose mother’s gestational weight gain was less than 7 kg and more than 16 kg, compared to children whose mother’s gestational weight gain was between 7 and 16 kg, it was more.

Also, there was significant relationship between WAZ with mother’s weight and height, so that mean weight and height of mothers with underweight child were lower than mothers with normal weight child.

There was also a statistically significant relationship between the HAZ with the economic status of the family, so that the stunting prevalence among children whose family income was not enough to live on compared to children whose family income was enough to live on, it was more (Table 4).

**Table 4:** Frequency distribution of anthropometric indices according to characteristics of mothers under study

Variables	Categories	WHZ n(%)			WAZ Underweight n(%)		HAZ Stunting n(%)	
		Wasting	Normal	Overweight	Yes	No	Yes	No
College education	*Yes*	12(6.5)	159(85.5)	15(8.1)	12(6.5)	174(93.5)	12(6.5)	174(93.5)
	*No*	10(5.5)	153(84.1)	19(10.4)	4(2.2)	178(97.8)	17(9.3)	165(90.7)
	p	0.696^a^			0.045^a^		0.304^a^	
Employment status	Housewife	16(5.7)	245(86.6)	22(7.8)	12(4.2)	271(95.8)	19(6.7)	264(93.3)
	University/School student	0(0.0)	1(100.0)	0(0.0)	0(0.0)	1(100.0)	0(0.0)	1(100.0)
	government employee	4(5.6)	59(81.9)	9(12.5)	2(2.8)	70(97.2)	7(9.7)	65(90.3)
	Self-employed	2(16.7)	7(58.3)	3(25.0)	2(16.7)	10(83.3)	3(25.0)	9(75.0)
	p	0.108^b^			0.168^b^		0.128^b^	
Ethnicity	Tork	20(7.1)	235(83.3)	27(9.6)	15(5.3)	267(94.7)	24(8.5)	258(91.5)
	Kord	2 (2.6)	68(88.3)	7(9.1)	0(0.0)	77(100.0)	4(5.2)	73(94.8)
	Fars	0(0.0)	9(100.0)	0(0.0)	1(11.1)	8(88.9)	1(11.1)	8(88.9)
	p	0.582^b^			0.060^b^		0.441^b^	
Family income sufficiency	*Yes*	14(4.7)	255(85.9)	28(9.4)	12(4.0)	285(96.0)	18(6.1)	279(93.9)
	*No*	8(11.3)	57(80.3)	6(8.5)	4(5.6)	67(94.4)	11(15.5)	60(84.5)
	p	0.124^b^			0.523^b^		0.008^a^	
Gestational weight gain (kg)*	< 7	2(3.4)	48(82.8)	8(13.8)	3(5.2)	55(94.8)	7(12.1)	51(87.9)
	7–12	13(6.3)	174(84.1)	20(9.7)	5(2.4)	202(97.6)	15(7.2)	192(92.8)
	13–16	1(1.7)	55(91.7)	4(6.7)	2(3.3)	58(96.7)	4(6.7)	56(93.3)
	> 16	6(14.0)	35(81.4)	2(4.7)	6(14.0)	37(86.0)	3(7.0)	40(93.0)
	p	0.160^b^			0.013^b^		0.649^b^	
Gestational age (week)*	37>	2(7.1)	26(929)	0(0.0)	1(3.6)	27(96.4)	3(10.7)	25(89.3)
	≥ 37	20(5.9)	286(84.1)	34(10.0)	15(4.4)	325(95.6)	26(7.6)	314(92.4)
	p	0.166^b^			1^b^		0.474^b^	
Breastfeeding status*	She is breastfeeding now	6(3.9)	132(86.3)	15(9.8)	6(3.9)	147(96.1)	12(7.8)	141(92.2)
	She was breastfeeding, but now has stopped breastfeeding	9(5.4)	144(85.7)	15(8.9)	7(4.2)	161(95.8)	13(7.7)	155(92.3)
	She did not breastfeed at all	7(14.9)	36(76.6)	4(8.5)	3(6.4)	44(93.6)	4(8.5)	43(91.5)
	p	0.145^b^			0.679^b^		0.964^b^	
Number of children	1	18(9.6)	151(80.7)	18(9.6)	12(6.4)	175(93.6)	17(9.1)	170(90.9)
	2	4(2.9)	121(88.3)	12(8.8)	4(2.9)	133(97.1)	7(5.1)	130(94.9)
	3	0(0.0)	37(92.5)	3(7.5)	0(0.0)	40(100.0)	4(10.0)	36(90.0)
	4	0(0.0)	3 (75.0)	1(25.0)	0(0.0)	4 (100.0)	1(25.0)	3(75.0)
	p	0.070^b^			0.245^b^		0.182^b^	
Age (Mean ± SD, yr)		32.18 ± 5.26	31.31 ± 6.15	32.38 ± 5.68	32.81 ± 5.11	31.40 ± 6.09	32.72 ± 5.33	31.35 ± 6.11
p		0.526^c^			0.363^d^		0.243^d^	
Weight (Mean ± SD, kg)		65.86 ± 6.61	70.25 ± 11.67	70.44 ± 10.02	65.06 ± 7.46	70.23 ± 11.41	68.58 ± 9.24	70.12 ± 11.48
p		0.208^c^			0.017^d^		0.482^d^	
Height (Mean ± SD, cm)		159.86 ± 5.23	162.19 ± 5.20	162.29 ± 6.21	159.68 ± 4.49	162.17 ± 5.33	162.68 ± 5.84	162.00 ± 5.27
p		0.135^c^			0.047^d^		0.509^d^	

*n* number, *SD* standard deviation, *yr* year, *kg* kilogram, *cm* centimeter; a, chi-square; b, fisher’s exact test; C, one-way ANOVA; d, independent T-test; *, in relation to the child under study

According to the study findings, there was significant relationship between WHZ with age range, so that the prevalence of wasting in children 0–12 months and overweight in 13–24 months were more than other age ranges. Also, there was significant relationship between WAZ and WHZ with gender, so that the prevalence of underweight and wasting were more in boys than girls and the prevalence of overweight was more in girls than boys.

The findings showed that prevalence of underweight and wasting were significantly more in children who are using powdered milk, as well as in children who had not started receiving complementary foods. Also, the prevalence of underweight and wasting were significantly more among children with a history of acute respiratory infections and diarrhea during the past 2 weeks than children with no history of infection (Table 5).

**Table 5:** Frequency distribution of anthropometric indices according to characteristics of children under study

Variables	Categories	WHZ n(%)			WAZ Underweight n(%)		HAZ Stunting n(%)	
		Wasting	Normal	Overweight	Yes	No	Yes	No
Age range	0–12	12(9.2)	114(87.7)	4(3.1)	8(6.2)	122(93.8)	7(5.4)	123(94.6)
	13–24	4(3.6)	93(83.0)	15(13.4)	4(3.6)	108(96.4)	10(8.9)	102(91.9)
	25–36	1(1.9)	45(84.9)	7(13.2)	1(1.9)	52(98.1)	6(11.3)	47(88.7)
	> 36	5(6.8)	60(82.2)	8(11.0)	3(4.1)	70(95.9)	6(8.2)	67(91.8)
	p	0.018^b^			0.654 ^b^		0.499 ^b^	
Gender	Male	16(8.2)	165(85.1)	13(6.7)	12(6.2)	182(93.8)	14(7.2)	180(92.8)
	Female	6(3.4)	147(84.5)	21(12.1)	4(2.3)	170(97.7)	15(8.6)	159(91.4)
	p	0.041^a^			0.048^a^		0.618^a^	
Birth order	First	17(8.8)	154(80.2)	21(11.0)	11(5.7)	181(94.3)	16(8.3)	176(91.7)
	Second	5(3.6)	125(89.3)	10(7.1)	5(3.6)	135(96.4)	10(7.2)	130(92.8)
	Third	0(0.0)	30(93.8)	2(6.2)	0(0.0)	32(100.0)	3(9.4)	29(90.6)
	Fourth	0(0.0)	4(100.0)	0(0.0)	0(0.0)	4(100.0)	0(0.0)	4(100.0)
	p	0.204^b^			0.418^b^		0.883^b^	
Interval from previous birth (month)	First birth	18(9.4)	155(80.7)	19(9.9)	12(6.2)	180(93.8)	14(7.3)	178(92.7)
	< 24	0(0.0)	17(89.5)	2(10.5)	0(0.0)	19(100.0)	3(15.8)	16(84.2)
	24–35	0(0.0)	29(87.9)	4(12.1)	1(3.0)	32(97.0)	4(12.1)	29(87.9)
	≥ 36	4(3.2)	111(89.5)	9(7.3)	3(2.4)	121(97.6)	8(6.5)	116(93.5)
	p	0.140^b^			0.367^b^		0.312^b^	
Type of milk consumed in the first 12 months of life	Breast milk	9(4.0)	197(86.8)	21(9.3)	8(3.5)	219(96.5)	16(7.0)	211(93.0)
	Powdered milk	8(11.4)	55(78.6)	7(10.0)	5(7.1)	65(92.9)	7(10.0)	63(90.0)
	Combination	5(7.0)	60(84.5)	6(8.5)	3(4.2)	68(95.8)	6(8.5)	65(91.5)
	p	0.222^b^			0.365^b^		0.626^b^	
Currently consumes breast milk	*Yes*	6(3.8)	138(87.9)	13(8.3)	6(3.8)	151(96.2)	12(7.6)	145(92.4)
	*No*	16(7.6)	174(82.5)	21(10.0)	10(4.7)	201(95.3)	17(8.1)	194(91.9)
	p	0.257^a^			0.669^a^		0.884^a^	
Currently consumes powdered milk	*Yes*	12(16.0)	61(81.3)	2(2.7)	7(9.3)	68(90.7)	4(5.3)	71(94.7)
	*No*	10(3.4)	251(85.7)	32(10.9)	9(3.1)	284(96.9)	25(8.5)	268(91.5)
	p	< 0.001^a^			0.018^a^		0.359^a^	
Currently consumes cow milk	*Yes*	10(6.2)	130(81.2)	20(12.5)	5(3.1)	155(96.9)	16(10.0)	144(90.0)
	*No*	12(5.8)	182(87.5)	14(6.7)	11(5.3)	197(94.7)	13(6.2)	195(93.8)
	p	0.156^a^			0.313^a^		0.186^a^	
Exclusive breastfeeding in the first 6-months of life	6 months are not over	2(4.2)	42(87.5)	4(8.3)	2(4.2)	46(95.8)	2(4.2)	46(95.8)
	*Yes*	11(5.4)	176(86.7)	16(7.9)	6(2.9)	197(97.1)	13(6.4)	190(93.6)
	*No*	11(9.4)	90(76.9)	16(13.7)	8(6.8)	109(93.2)	15(12.8)	102(87.2)
	p	0.240^a^			0.249^b^		0.157^a^	
Start receiving complementary foods (month)	Not started	7(12.7)	48(87.3)	0(0.0)	7(12.7)	48(87.3)	4(7.3)	51(92.7)
	< 4	1(5.9)	16(94.1)	0(0.0)	0(0.0)	17(100.0)	0(0.0)	17(100.0)
	4–6	3(3.2)	82(88.2)	8(8.6)	3(3.2)	90(96.8)	9(9.7)	84(90.3)
	≥ 6	11(5.4)	166(81.8)	26(12.8)	6(3.0)	197(97.0)	16(7.9)	187(92.1)
	p	0.008^b^			0.031^b^		0.725^b^	
History of acute respiratory infection in the last two weeks	*Yes*	3(20.0)	10(66.7)	2(13.3)	3(20.0)	12(80.0)	3(20.0)	12(80.0)
	*No*	19(5.4)	302(85.6)	32(9.1)	13(3.7)	340(96.3)	26(7.4)	327(92.6)
	p	0.035^b^			0.022^b^		0.105^b^	
History of diarrhea in the last two weeks	*Yes*	5(18.5)	22(81.5)	0(0.0)	4(14.8)	23(85.2)	4(14.8)	23(85.2)
	*No*	17(5.0)	290(85.0)	34(10.0)	12(3.5)	329(96.5)	25(7.3)	316(92.7)
	p	0.008^b^			0.023^b^		0.252^b^	
Hospitalization history in the last 1 month	*Yes*	0(0.0)	3(100.0)	0(0.0)	0(0.0)	3(100.0)	1(33.3)	2(66.7)
	*No*	22(6.0)	309(84.7)	34(9.3)	16(4.4)	349(95.6)	28(7.7)	337(92.3)
	p	1^b^			1^b^		0.219^b^	
Hospitalization history in the last 3 month	*Yes*	0(0.0)	7(100.0)	0(0.0)	0(0.0)	7(100.0)	1(14.3)	6(85.7)
	*No*	22(6.1)	305(84.5)	34(9.4)	16(4.4)	345(95.6)	28(7.8)	333(92.2)
	p	1^b^			1^b^		1^b^	
birth (Mean ± SD,gr) Weight at		3029.54 ± 552.19	3184.03 ± 434.92	3227.94 ± 441.62	3018.75 ± 610.97	3186.13 ± 434.34	3134.48 ± 388.93	3182.65 ± 448.36
p		0.229^c^			0.295^d^		0.336^d^	
Height at birth (Mean ± SD, cm)		49.62 ± 2.17	49.80 ± 1.98	49.70 ± 1.31	49.67 ± 2.59	49.78 ± 1.90	49.41 ± 2.02	49.81 ± 1.92
p		0.896^c^			0.823^d^		0.288^d^	

*n* number, *SD* standard deviation, *gr* gram, *cm* centimeter; a, chi-square; b, fisher’s exact test; C, one-way ANOVA; d, independent T-test

According to the study findings, there was a significant relationship between the maternal nutrition literacy with anthropometric indices of under study children, so that the mean score of nutritional literacy among the mothers with underweight, wasting and stunting children was lower than the mothers with normal children (Table 6).

**Table 6:** Comparison of mothers’ mean score of nutritional literacy based on anthropometric indices

Anthropometric indices	Subcategories		Mean ± SD	p
weight-for-height (WHZ)	wasting		23.45 ± 4.42^a^	0.004^b^
	Normal		26.19 ± 4.46^a^	
	Overweight		24.47 ± 5.29	
weight-for-age (WAZ)	Underweight	Yes	23.31 ± 5.77	0.023^c^
		No	25.98 ± 4.51	
height t-for-age (HAZ)	Stunting	Yes	23.03 ± 5.13	0.001^c^>
		No	26.11 ± 4.47	

a, statistically significant difference between the two groups based on the Bonferroni correction method. b, one-way ANOVA; c, independent T-test

## Discussion

Based on the study findings, prevalence of wasting, underweight, stunting, and overweight, were respectively 6, 4.3, 7.9, and 9.2% among the studied children less than 5 years old. In a study conducted by Farrokh-Eslamlou et al., prevalence of wasting, underweight and stunting in less than 5 years old children of West Azerbaijan were respectively 5.7, 3.7, and 8.7% that these findings are almost in line with the findings of the present study [14].

Various studies have been conducted on the prevalence of malnutrition and investigation of anthropometric indices among Iranian children, but most of these studies are at the provincial level or a city, and studies conducted at the national level are limited [15]. Also, the estimates of the prevalence of malnutrition based on anthropometric indices in variety studies is different, which makes it difficult to compare the findings of present study with the findings of these studies. For example in the study conducted by Houshiar Rad et al., the prevalence of wasting, underweight, stunting, and overweight were obtained respectively 4.5, 7.6, 13.1, and 5.2% among the Iranian children less than 5 years old [16]. In the study conducted by Mohseni et al., the prevalence of wasting, underweight, and stunting were respectively 7.8, 10.5, and 12.4% [17]. Also, in the study conducted by Motedayen et al., the prevalence of wasting, underweight and stunting were respectively 6, 7 and 11% among the Iranian children less than 5 years old.

According to the report, in 2020 globally, 149.2 million children (22%) under the age of 5 were stunted, 45.4 million (6.7%) were wasted, and 38.9 million (5.7%) were overweight [18]. Also, in the study conducted by Ssentongo et al., in low- and middle-income countries, the prevalence of wasting, underweight and stunting among children under 5 years of age were 6.3, 13.7 and 29.1%, respectively [19].

Comparing the findings of the present study with the findings of the other studies [16–19], indicates the improvement of the situation of children in Urmia in terms of wasting, underweight and stunting over time, but it must be mentioned that although the percentage of malnutrition based on wasting, underweight and stunting indices is lower than other reports, but this problem still strongly remains. In particular, stunting index which has a higher percentage than wasting and underweight indices in this research. Stunting index, while indicating chronic malnutrition at the individual level, it is also one of the strongest indicators of hunger and poverty and can be a warning factor [4]. Therefore, it is recommended in nutrition improvement programs, in addition to improving the quantity and quality of nutrition and health services, poverty alleviation programs and the development of deprived areas must be implemented.

Moreover, according to this research’s findings, the prevalence of overweight among children under 5 years of age in Urmia is higher than other reports, for instance, in comparison with the study of Houshiar Rad et al., the prevalence of overweight among children under 5 years of age in Urmia has almost doubled [16] or almost tripled in comparison with the study of Emamian et al. [20]. Currently, there is an international agreement to achieve a rate of less than 5% for wasting, less than 6% for overweight and about 14.7% for stunting by 2025 [21]. Achieving these purposes needs designing and implementing various interventions, especially educational and nutritional interventions among children and mothers. In order to increase the effectiveness of these interventions, first, the factors influencing anthropometric indices must be determined in order to concentrate our interventions on those factors which are significantly related with these indices and can be changed via the intervention.

According to this research’s findings, one of the factors which had a statistically significant relationship with anthropometric indices, was the maternal nutritional literacy, so that the mean score of nutritional literacy among mothers with wasting, underweight and stunting child was lower than mothers with normal child. In line with the findings of the present study, in studies conducted by Fadare et al. [7], Oly-Alawuba et al. [9], and Saaka [8], there was a positive and significant relationship between maternal nutritional literacy with WAZ, HAZ, and WHZ indices of children, which confirm the findings of this study. In contrast to this study’s findings, in study conducted by Wanjihia et al. [22], there was no significant relationship between maternal nutritional literacy and WAZ, HAZ and WHZ indices of children. So, authors of article stated two reasons for justifying this finding: 1) In examining the relationship between maternal nutritional literacy and anthropometric indices, confounding variables were not controlled, 2) and also assessing the nutritional knowledge of mothers/caregivers was not based on local context or foods available locally [22].

According to this research’s findings and other research conducted [7–9], it can be concluded that maternal nutritional knowledge is still a fundamental factor and must be regarded in efforts aimed at enhancing anthropometric indices and eradicating malnutrition among children. Nutrition education is one of the interventions which can help enhance the maternal nutritional literacy. Therefore, designing and implementing educational programs applying existing theories and models in health education science with the purpose of enhancing mothers’ nutritional knowledge is recommended, implementing such interventions can finally lead to enhanced nutritional status and anthropometric indices of children [22]. But, it must be mentioned that maternal nutritional knowledge is not the only determinant of nutritional status and anthropometric indices of children, other factors may have an effect which must be determined and in efforts aimed at enhancing anthropometric indices among children, they must also be regarded.

Based on this research’s findings, the prevalence of underweight among children whose mothers had a college education was higher than children whose mothers did not have a college education and also among the children whose mother’s gestational weight gain was less than 7 kg and more than 16 kg, compared to children whose mother’s gestational weight gain was between 7 and 16 kg. Moreover, the prevalence of stunting among children whose family income was less than adequate living was higher than children whose family income was sufficient.

Based on this research’s findings, it can be concluded that high education and college education do not guarantee nutritional status and optimal weight in children and educational interventions related to children’s nutrition, even among mothers with college education, should be implemented. Thus, conflicting results were reported considering the relation between mother’s education level and child anthropometric indices. For instance, in the research conducted by Anzar et al., the results indicated that there was no significant relationship between maternal education level and WAZ and HAZ indices [23], while in the study conducted by Makoka et al., the results indicated that there was a positive and significant relationship between maternal education level with WAZ, HAZ and WHZ indices [24]. Therefore, more research are recommended to provide more correct results in this field.

Furthermore, according to this research’s findings, the mother’s gestational weight gain less than 7 and over 16 kg is a risk factor for underweight in children under 5 years of age, which it is required to be regarded and in preconception and prenatal education, mothers must be encouraged to have a normal weight gain and proportionate to their BMI during their pregnancy.

This research’s findings indicated that the prevalence of stunting among children whose family income was lower than the adequate level of life is higher than children whose family income was sufficient, this result was not far from expectation and is in line with the results of many studies in this field [22, 25]. Even in some research, it was noted that family income is a stronger factor in identifying children’s nutritional status than mothers’ nutritional knowledge [22]. Poverty and illiteracy are the top two leading causes of malnutrition and in nutrition improvement programs, priority should be given to disadvantaged, vulnerable and low-income groups [26].

Based on this research’s findings, the prevalence of wasting in the children 0–12 months was more than other age ranges. Consistent with this finding, in the study conducted by Aguayo et al., wasting prevalence was significantly higher among the children 0–11 months. The cause of this finding was attributed to poor complementary feeding practices in the first year of life which were not aligned with internationally agreed-upon guidance. Global and national policy recommends that infants aged 0–5 months be exclusively breast-fed, with no other fluids or foods given, not even water, while children aged 6–23 months should be fed age-appropriate soft, semi-solid or solid complementary foods while breast-feeding continues [27].

Also, the prevalence of underweight and wasting in males was higher than that of girls, which is in line with the study conducted by Ahmadi et al. (5). Moreover, among children with a history of acute respiratory infection and diarrhea during the past 2 weeks, the prevalence of underweight and wasting was significantly higher than among children without a history of these diseases, this result is not far from expectation and is in line with the results of other studies [5, 25].

Moreover, the prevalence of underweight and wasting was significantly higher among children who were currently consuming powdered milk and children who had not started complementary foods until after 6 months of age. Therefore, it is recommended to regard all these factors when designing and implementing interventions to enhance nutritional status and anthropometric indices of children, and focus our interventions more on mothers and children who have these risk factors, including mothers with low nutritional literacy, mothers whose gestational weight gain was not normal, children whose family income is low, children aged 0–12 months, boy child, children with a history of disease, children who consume powdered milk and children who have not started complementary foods at the right time.

One of the limitations of the present study was that the data related to maternal nutritional literacy, socio-economic and demographic variables were collected by a self-report method, so there was possibility that participants may have not given real answers to the questions.

## Conclusions

The findings of the present study show that the Urmia children status in has improved in terms of wasting, underweight, and stunting over time, but it should be noted that this problem strongly remains. In particular, stunting index which has a higher percentage than wasting and underweight indices in this research. Moreover, the prevalence of overweight among children under 5 years of age in Urmia is higher than other reports. Therefore, designing and implementing various interventions, especially educational interventions in the field of nutrition, with the purpose of enhancing nutritional status and anthropometric indices of children, is recommended among mothers. It is also suggested to focus more on those factors which have a significant relationship with anthropometric indices to increase the effectiveness of these interventions. According to this research’s findings, maternal nutritional literacy level, mother’s education level, gestational weight gain, family income, child’s gender, history of acute respiratory infection and diarrhea, powdered milk consumption and lack of starting complementary food at the right time are among the factors that are significantly related with child anthropometric indices and in efforts aimed at enhancing anthropometric indices and eradicating malnutrition among children must be considered.

## Data Availability

The datasets used and/or analysed during the current study are available from the corresponding author on reasonable request.

## Abbreviations

MNL: Maternal Nutritional Literacy
EINLA: Evaluation Instrument of Nutrition Literacy on Adults
WAZ: Weight-for-Age Z-Score
HAZ: Height-for-Age
WHZ: Weight-for-Height
WHO: World Health Organization
IR: Iran
UMSU: Urmia University of Medical Sciences
REC: Research Ethics Committee
SPSS: Statistical Package for the Social Sciences
ANOVA: Analysis of Variance
kg: Kilogram
BMI: Body mass index
n: Number
SD: Standard Deviation
yr: Year
cm: Centimeter
gr: Gram
